# Hypertriglyceridemia and atherosclerosis in apolipoprotein A5 mutant rabbits generated using CRISPR/Cas9

**DOI:** 10.1016/j.jlr.2026.101151

**Published:** 2026-09-22

**Authors:** Yiwen Zha, Lin Feng, Ting Zhang, Kunning Yan, Xingyi Wang, Yong Cheng, Jingyan Liang

**Affiliations:** 1 Northern Jiangsu People's Hospital Affiliated to Yangzhou University, Yangzhou, Jiangsu, China; 2 Northern Jiangsu People's Hospital, Yangzhou, Jiangsu, China; 3 School of Basic Medical Sciences & School of Public Health, Faculty of Medicine, Yangzhou University, Yangzhou, China; 4 Animal Husbandry and Veterinary College, Jiangsu Vocational College of Agriculture and Forestry, Jurong, Jiangsu, China; 5 College of Veterinary Medicine, Yangzhou University, Yangzhou, Jiangsu, China; 6 Jiangsu Co-Innovation Center for Prevention and Control of Important Animal Infectious Disease and Zoonoses, Yangzhou, China; 7 Jiangsu Key Laboratory of Integrated Traditional Chinese and Western Medicine for Prevention and Treatment of Senile Disease, Yangzhou University, Yangzhou, Jiangsu, China

**Keywords:** hypertriglyceridemia, atherosclerosis, apolipoproteinA5, rabbits, residual cardiovascular risk

## Abstract

Apolipoprotein A5 (APOA5) plays an important role in regulating plasma triglycerides (TGs). Most in vivo data, however, derive from murine models whose lipoprotein metabolism diverges substantially from humans, and no rabbit *APOA5* loss-of-function model has previously been described. Microinjection of Cas9 mRNA and two exon-2-targeting single-guide RNAs into rabbit zygotes yielded 10 founders carrying biallelic loss-of-function alleles, and with an approximately 90% reduction in plasma APOA5 protein. On chow, *APOA5* mutant rabbits developed a stable atherogenic dyslipidemia with selectively elevated plasma TGs and reduced HDL-C, both more severe in male than in female mutants. The dyslipidemia was paralleled by accumulation of apolipoprotein B-containing lipoproteins, an enlarged VLDL distribution by fast-protein liquid chromatography, and reduced pre-heparin plasma activities of lipoprotein lipase and hepatic lipase together with correspondingly lower plasma FFAs. Mutants also exhibited elevated plasma IL-1β, TNF-α and CCL2 with a selective expansion of circulating monocytes, granulocytes, and platelets, indicating a low-grade systemic inflammatory state already under chow conditions. After 8 weeks on a high-fat, cholesterol-enriched diet, mutants developed severe combined dyslipidemia, extensive aortic atherosclerosis with macrophage- and smooth-muscle-rich plaques, and marked hepatic steatosis. In conclusion, biallelic disruption of rabbit *APOA5* produces a translationally relevant phenotype that recapitulates principal features of human APOA5 deficiency. The *APOA5* mutant rabbit thus provides a tractable large-animal platform for studying residual cardiovascular risk and for evaluating emerging TG-lowering therapeutics.

## Graphical abstract

*APOA5* mutant rabbits, generated by CRISPR/Cas9 targeting of exon 2, exhibit APOA5 deficiency and a corresponding decrease in plasma lipoprotein lipase (LPL) and hepatic lipase (HL) activities. The resulting impairment of triglyceride-rich lipoprotein hydrolysis causes accumulation of VLDL and chylomicron-derived particles, reduced plasma FFAs, and decreased HDL-C, producing spontaneous hypertriglyceridemia on chow that is converted into aggravated aortic atherosclerosis under a high-fat diet. Because rabbit and human lipid metabolism are closely related, the *APOA5* mutant rabbit serves as a translational platform for studying hypertriglyceridemia-driven residual cardiovascular risk and for evaluating new triglyceride-lowering therapeutics.

**Figure:**
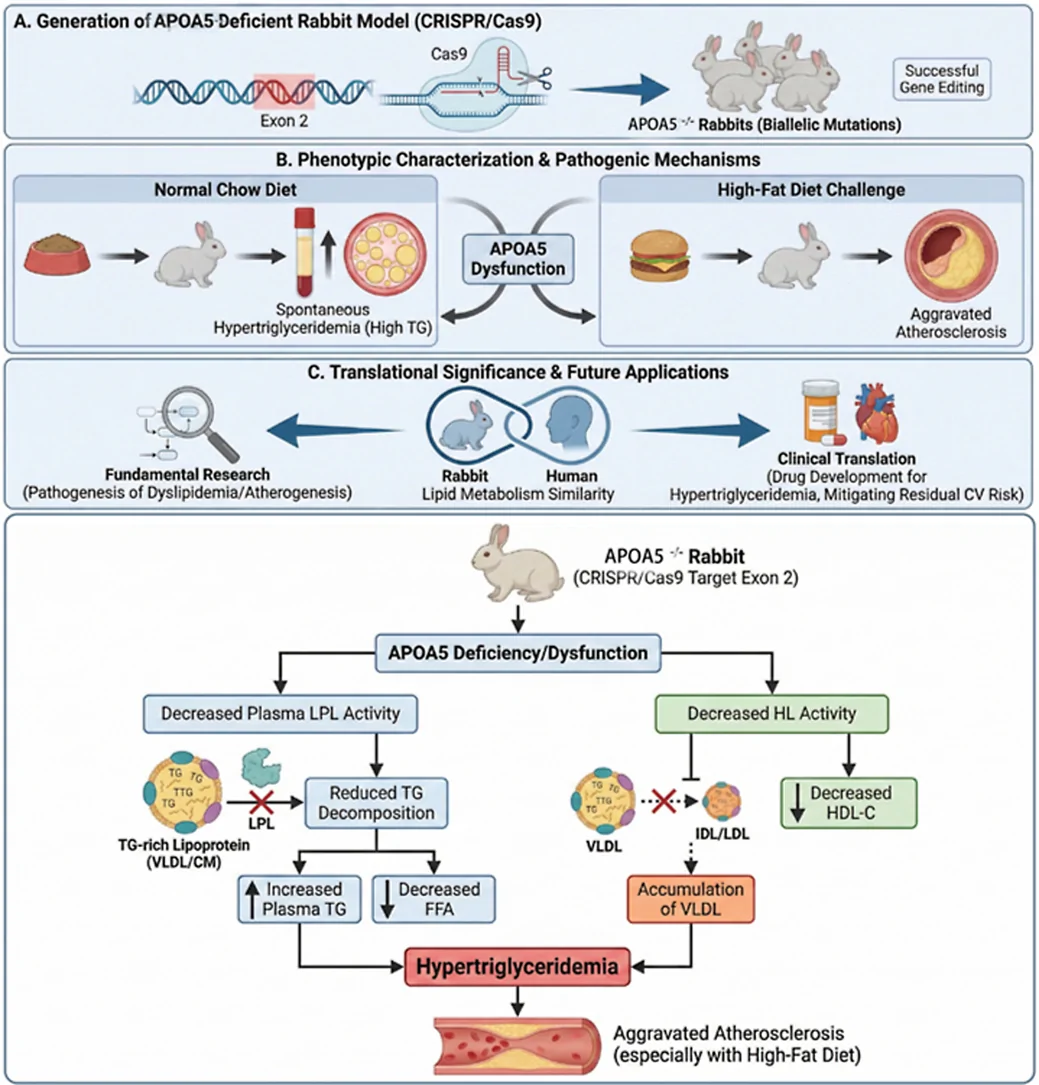

Despite the clinical success of LDL-cholesterol-lowering therapy, individuals on guideline-recommended statin and PCSK9 regimens continue to suffer atherosclerotic cardiovascular events at substantial rates. Triglyceride (TG)-rich lipoproteins (TRLs) and their remnants have emerged as a principal source of this residual risk, with both observational and Mendelian randomization data demonstrating a causal contribution to atherogenesis that is at least partly independent of LDL-cholesterol (1, 2, 3). Apolipoprotein A5 (APOA5), a 39-kDa hepatic protein originally identified through comparative genomic sequencing (4), is among the most potent endogenous suppressors of plasma TG. Loss-of-function variants in *APOA5* are associated with a 2- to 4-fold elevation in TG and a 2.2-fold elevation in the risk of myocardial infarction in large exome-sequencing studies (5), while pharmacological neutralization of circulating APOA5 in mice produces hypertriglyceridemia within hours by destabilizing endothelial-bound LPL (6).

Mechanistically, APOA5 lowers TG predominantly by binding to and disassembling the ANGPTL3/8 complex, thereby protecting LPL from inhibition and supporting capillary-bound LPL-mediated TG hydrolysis (7, 8, 9). APOA5 has also been shown to interact with members of the LDL-receptor family (10) and with heparan-sulfate proteoglycans (11), and in mouse models to restrain hepatic large-VLDL particle assembly through an SREBP-1c-dependent pathway (12). *In vivo* dissection of these consequences has, however, been hampered by the limitations of murine genetics. Mice secrete both APOB100- and APOB48-containing VLDL, exhibit a predominantly HDL-rich plasma profile, and lack cholesteryl-ester transfer protein activity features that diverge substantially from human physiology. Rabbits, by contrast, secrete VLDL containing APOB100 only, possess plasma CETP activity and develop atherosclerotic plaques whose architecture parallels that of human lesions, making them a long-established model for translational lipoprotein research (13, 14, 15). Recent reports of CRISPR-engineered hypertriglyceridemic hamsters (16, 17) highlight the value of mammalian models beyond the mouse that more faithfully recapitulate human chylomicronemia and its complications, but no equivalent rabbit *APOA5* model has previously been described. Importantly, human and rabbit APOA5 share 84% sequence identity, both genes comprising four exons and three introns, and the genomic neighborhood at the *APOA5/A4/C3/A1* locus is conserved (18).

Here, we describe the generation, characterization, and pathological consequences of *APOA5*-mutant rabbits maintained on chow or on a high-fat, cholesterol-enriched diet, and we evaluate how the resulting metabolic profile relates to plasma APOA5 protein concentration, lipase activities, and atherosclerotic burden.

## Materials and methods

### Animals

New Zealand White rabbits were bred and housed in the Animal Genetic Engineering Laboratory at Yangzhou University at 24 °C, 55% relative humidity and on a 12 h light/12 h dark cycle, with ad libitum access to water and twice-daily feeding. Until week 20, rabbits received a chow diet (NC); thereafter, the surviving cohort was challenged with a high-fat diet (HFD) (TP 2R301, Trophic, Ltd, Nantong, China) for 8 weeks. All animal procedures were approved by the Animal Care and Use Committee of Yangzhou University and conformed to the National Research Council Guide for the Care and Use of Laboratory Animals.

We initially obtained 10 WT and 10 APOA5-mutant rabbits, all age-matched. One mutant (V10) died on the day of birth from a maternally inflicted injury, and only ear tissue was retained for genotyping. A second mutant (V2) died unexpectedly at 7 weeks of age. Two additional mutants (V7 and V8) were subsequently excluded from pathological analysis and reserved for breeding. Thus, the final cohort for terminal pathological assessments comprised 10 WT controls and 6 mutant rabbits.

### Anesthesia, analgesia, and euthanasia

For all surgical and terminal procedures, rabbits were fasted 4–6 h and anaesthetized by intramuscular ketamine (13 mg/kg) plus xylazine (5 mg/kg). At study endpoint, animals were rendered deeply unconscious by the previous induction regimen and euthanized by exsanguination via cardiac puncture, in accordance with AVMA guidelines for laboratory rabbits.

### Cas9 mRNA/single-guide RNA (sgRNA) preparation and zygote microinjection

The genomic sequence of the rabbit APOA5 gene was obtained from the NCBI database. Using this sequence, single-guide RNAs (sgRNAs) were designed to target exon 2 of the gene with the CRISPR Design tool (http://crispr.mit.edu). Two specific sgRNAs (depicted in Supplemental Fig. S1A) were ultimately selected for use in generating the mutant loci.

Embryos were obtained from sexually mature female rabbits (over 6 months old) mated with fertile males. Donor females were superovulated with 60 IU of pregnant mare serum gonadotropin, followed by an injection of 10 IU human chorionic gonadotropin 72 h later. Immediately after human chorionic gonadotropin administration, the donors were mated with male rabbits. Embryos were collected by flushing the oviducts with PBS 18–20 h after mating. A mixture of Cas9 mRNA (33 ng/μl) and the two sgRNAs (20 ng/μl each) was microinjected into the cytoplasm of the zygotes under a Leica inverted microscope. The injected embryos were briefly cultured in M2 medium microdrops under mineral oil at 38°C in a 5% CO_2_ atmosphere for 30 min. Finally, 10–15 viable zygotes were surgically transferred into the oviducts of each pseudopregnant recipient rabbit. Embryo-transfer outcomes are summarized in Supplemental Fig. S1B.

### Genotyping and off-target assessment

Genomic DNA was isolated from ear-tag biopsies by proteinase-K digestion and phenol/chloroform extraction. The targeted *APOA5* exon 2 was amplified with F1 (5′-GGGTGGGAACTGAGACAGC-3′) and R1 (5′-GAGGTAGCAGCGAGCGTAG-3′) and Sanger-sequenced. To resolve compound heterozygosity, PCR products were cloned into pMD™ 19-T (Takara Bio, Inc., Japan) and a minimum of eight colonies per founder were sequenced. The five highest-scoring potential off-target sites for each sgRNA were PCR-amplified with the primers in Supplemental Fig. S1C and Sanger-sequenced; no edits were detected at any off-target locus in any founder.

### Plasma lipid and apolipoprotein analyses

EDTA plasma was collected after 12 h of fasting. The total cholesterol (TC), TG, LDL-C, and HDL-C levels in plasma were measured enzymatically using commercially available kits (Jiancheng Bioengineering Institute, Nanjing, China). Western blotting was used to detect plasma Apob, plasma APOA5, and liver APOA5. The following primary antibodies were used: goat anti-Apob (Rockland, Limerick, PA), and a goat polyclonal antibody against APOA5 (HUABIO, Hangzhou, China), which was custom-generated by immunizing goats with a specific peptide antigen. The secondary Abs were HRP-conjugated donkey anti-goat IgG (Jackson Immuno Research Laboratories, West Grove, PA).

### Plasma lipoprotein profiling

For agarose-gel lipoprotein electrophoresis, 2 μl of plasma was applied to a 1% agarose gel (Helena Laboratories, Saitama, Japan), electrophoresed in barbital buffer, and stained with Fat Red 7B for neutral-lipid visualization. For fast-protein liquid chromatography (FPLC), 200 μl of pooled plasma was applied to a tandem Superose-6 10/300 Gl column (Cytiva, Uppsala, Sweden) equilibrated in PBS containing 1 mM EDTA, eluted at 0.4 ml/min, and 0.4 ml fractions were collected.

### Lipase activity and plasma-FFAs

Plasma activities of LPL and HL were measured without prior heparin injection of the animals, so as to capture the steady-state circulating (pre-heparin) pool. Whole blood was collected from the central ear artery into K_2_EDTA tubes as described previously; plasma was separated and aliquoted. LPL activity, HL activity and plasma FFA concentration were measured using separate, lipase-specific colorimetric kits from Solarbio Life Science (Beijing, China; catalog numbers BC2440, BC2380 and BC0590), strictly according to the manufacturer's instructions.

### Plasma inflammatory mediators and whole-blood counts

IL-1β and IL-6 were measured by rabbit-specific ELISA (Elabscience Biotechnology Co., Wuhan, China; catalog numbers E-EL-RB0027 and E-EL-RB0009). TNF-α and CCL2 were measured by rabbit-specific ELISA (Cusabio Biotech, Wuhan, China; CSB-E06998Rb and CSB-E07432Rb) per the manufacturers' protocols. Whole-blood counts (white blood cell count, monocytes, lymphocytes, granulocytes, platelet count, plateletcrit) were performed on EDTA-anticoagulated blood using a Mindray BC-2800Vet auto-hematology analyzer (Mindray Medical, Shenzhen, China).

### Histopathology, immunohistochemistry, and lesion quantification

After 8 weeks of HFD, rabbits were anaesthetized and sacrificed according to the methods described previously, and then aortic trees and hearts were excised. The aortas were opened longitudinally, postfixed for 24 h, and stained en face with Sudan IV (Solarbio, Beijing, China). The Sudan-positive area was quantified in Image-Pro Plus 6 (Media Cybernetics, Inc.) and expressed as a percentage of total aortic surface. For lesion architecture, the aortic root and the left coronary artery were paraffin-embedded, serially sectioned (5 μm), and stained with hematoxylin-eosin, Masson's trichrome, and immunohistochemically stained with monoclonal antibodies against either macrophages (Mϕ) (clone: RAM11, Dako, Carpinteria, CA) or α-smooth muscle actin for smooth muscle cells (clone: HHF35, Dako, Carpinteria, CA). For α-SMA staining, adjacent sections were incubated with isotype-matched IgG in place of the primary antibody as negative controls.

### Histological and biochemical analysis of liver

Liver pieces from the left lobe were fixed in 10% formalin for 48 h, paraffin-embedded, and hematoxylin-eosin-stained. For Oil Red O (ORO) staining, freshly excised liver was cryoprotected in 30% sucrose, embedded in OCT, sectioned at 10 μm on a Leica CM1950, fixed in 4% paraformaldehyde, stained in 0.5% ORO/isopropanol, and counterstained with hematoxylin. Plasma alanine aminotransferase and aspartate aminotransferase activities were measured with colorimetric kits from the Nanjing Jiancheng Bioengineering Institute (Nanjing, China; catalog numbers C009-2 and C010-2) according to the manufacturer's instructions.

## Results

### Generation of *APOA5* mutant rabbits

Rabbits offer a closer recapitulation of human plasma lipoprotein metabolism than rodents (13), yet a rabbit model of *APOA5* loss-of-function has not been described. We therefore set out to generate such a model by targeting exon 2 of the rabbit *APOA5* locus. A total of 82 microinjected zygotes were transferred to five pseudopregnant recipients (13–18 embryos each), yielding 10 live founders (V1–V10; Supplemental Fig. S1B). One founder (V10) was lost within 24 h of birth due to a maternally inflicted injury, with ear tissue retained for genotyping, and a second founder (V2) died unexpectedly at 7 weeks of age.

Sanger sequencing of *APOA5* exon 2 PCR products from all 10 founders revealed overlapping chromatographic peaks indicative of compound heterozygous editing (Supplemental Fig. S2A); TA-cloning resolved the underlying alleles as a combination of small deletions, insertions and base substitutions (Supplemental Fig. S2B), with the translated amino-acid sequences predicted in Supplemental Figure S2C. Two founders (V1♀ and V6♂) carried in-frame deletion/insertion mutations confined to the signal-peptide-encoding region; two further founders (V5♀ and V8♀) carried frameshift mutations leading to premature termination, and the remaining founders carried one allele of each type. Sanger sequencing of the five highest-ranked potential off-target sites for each sgRNA returned no detectable edits in any founder (Supplemental Fig. S1C), arguing against off-target activity of the editing system. These genetically characterized *APOA5* mutants therefore provided the foundation for the systematic phenotypic characterization that follows.

### Plasma APOA5 protein and lipid profile of *APOA5* mutant rabbits

Because biallelic editing of exon 2 was confirmed at the genomic level in every founder, we next asked whether this lesion translates into a functional reduction of circulating APOA5 protein and into a measurable plasma lipid phenotype. Western blotting of plasma sampled at 6 weeks of age revealed near-complete loss of mature APOA5 in mutants, with densitometric quantification corresponding to an approximately 90% reduction in circulating APOA5 relative to WT littermates (Fig. 1A, B). Enzymatic plasma lipid profiling at the same time-point disclosed a selective and pronounced elevation of TGs together with a fall in HDL-C; total cholesterol and LDL-cholesterol did not differ between genotypes on the chow diet (Fig. 1C–F). Within the mutant group, male rabbits exhibited higher plasma TGs than their female littermates (Fig. 1G) and a reciprocally lower plasma HDL-C (Supplemental Fig. S3A), in keeping with the recognized sex-dependent modulation of plasma lipid metabolism by estrogens (19, 20).

**Fig. 1:**
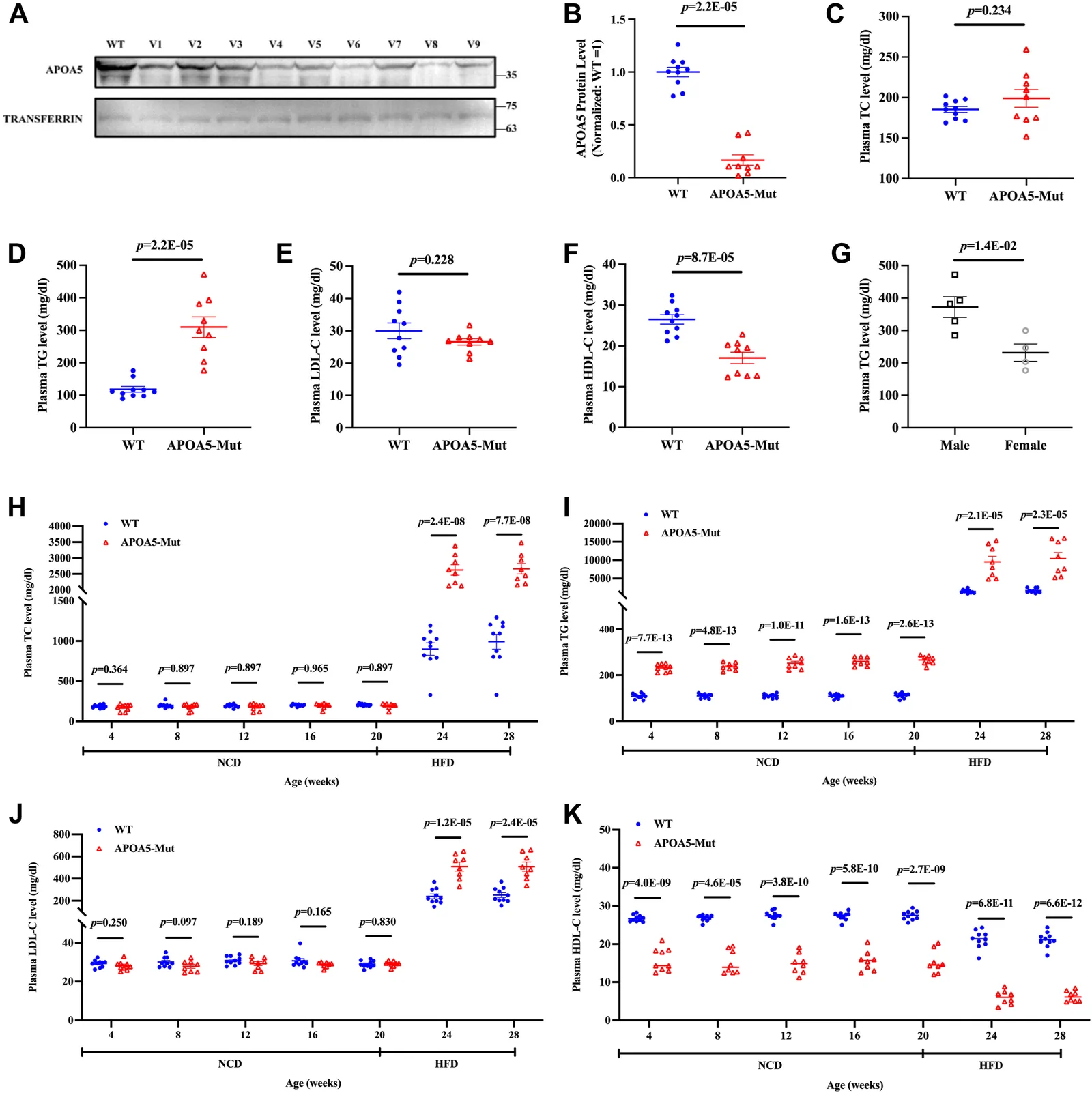
Plasma APOA5 protein and lipids in *APOA5* mutant rabbits. A: Representative Western blot of plasma APOA5 from WT and APOA5-Mut rabbits at 6 weeks of age. B: Quantification of A. C–F: Plasma TC, TG, LDL-C, and HDL-C at 6 weeks of age (WT, n = 10; APOA5-Mut, n = 9). G: Plasma TG at 6 weeks stratified by sex within the *APOA5*-mutant group (male, n = 5; female, n = 4). H–K: Longitudinal plasma TC, TG, LDL-C, and HDL-C at 4, 8, 12, 16, 20, 24, and 28 weeks of age. Data are mean ± SEM; statistical tests and exact *P*-values are annotated on each panel. APOA5, apolipoprotein A5; TG, triglyceride.

Repeated bleeds of the same animals at weeks 4, 8, 12, 16, 20, 24, and 28 placed this 6-weeks snapshot in a longitudinal context (Fig. 1H–K). Throughout the chow phase, the hypertriglyceridemia and reduced HDL-C of *APOA5* mutant rabbits were stable, whereas total and LDL-cholesterol remained comparable between the two groups. The sex-dependent severity observed at 6 weeks was maintained across this longitudinal window: in the mutant group, males consistently had higher plasma TGs (Supplemental Fig. S3C) and lower HDL-C (Supplemental Fig. S3B) than females. Following introduction of a high-fat, cholesterol-enriched diet at week 20, both genotypes developed dyslipidemia, but the response of mutants was disproportionately amplified: by week 28, plasma TGs, total cholesterol, and LDL-cholesterol were each substantially higher in mutants than in WT controls, while HDL-C declined further. Together, these data establish *APOA5* mutant rabbits as a model of severe, diet-amplified atherogenic dyslipidemia with reproducible sex-dependent severity.

### Plasma apolipoprotein B distribution and lipase activities

The dyslipidemia documented previously predicts changes in the plasma distribution of apoB-containing lipoproteins and in the activity of the principal plasma lipases that act on TRLs (7). We therefore examined these readouts in the same chow-diet cohort. Western blotting of plasma detected the expected dual APOB pattern in both genotypes, but quantification revealed accumulation of both APOB100 and APOB48 in mutants (Fig. 2A–C); because rabbit VLDL contains only APOB100, the rise in plasma APOB48 reflects accumulation of intestine-derived chylomicrons and chylomicron remnants rather than hepatic VLDL particles. Agarose-gel electrophoresis of plasma lipoproteins showed a shift of the mutant lipoprotein pattern toward the pre-β region, with a reciprocal contraction of the α band (Fig. 2D), consistent with accumulation of TRLs alongside HDL depletion. FPLC fractionation of pooled plasma further localized the major TG-rich peak in mutants to one fraction earlier than in WT controls (Fig. 2E), indicating an enlarged VLDL particle distribution.

**Fig. 2:**
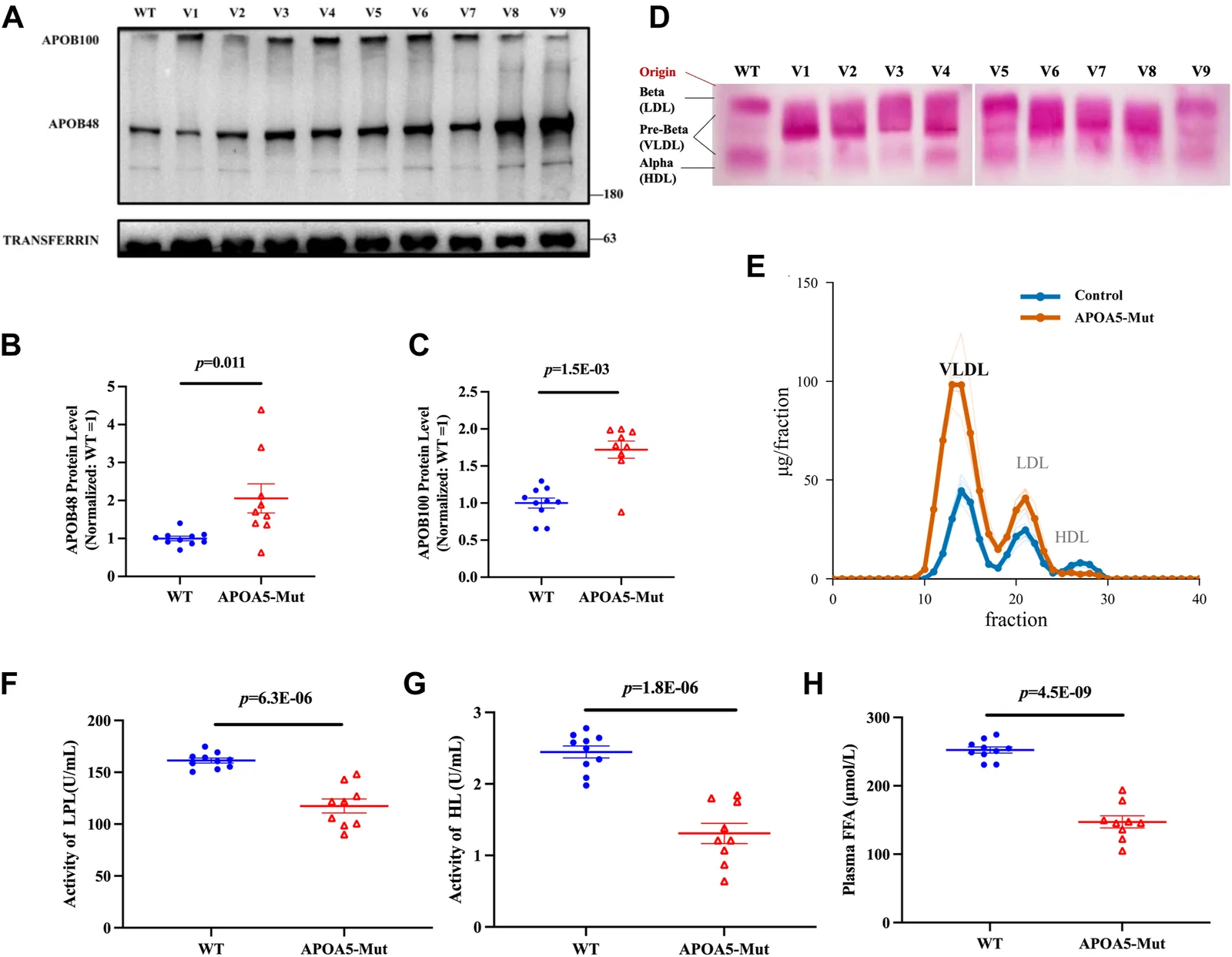
Apolipoprotein-B-containing lipoprotein accumulation and reduced plasma lipase activity in *APOA5* mutant rabbits. A: Representative Western blot of plasma APOB100 and APOB48 at 18 weeks of age. B–C: Quantification of APOB48 (B) and APOB100 (C) (WT, n = 10; APOA5-Mut, n = 9). D: Agarose-gel electrophoresis of plasma lipoproteins (2 μl/lane) stained with Fat Red 7B. E: FPLC fractionation of plasma on tandem Superose-6 columns; TG concentration in each 0.4-mL fraction is plotted. F–H: Plasma LPL activity (F), HL activity (G), and plasma FFA (H) measured at 6 weeks of age. Data are mean ± SEM; statistical tests and exact *P*-values are annotated on each panel. APOA5, apolipoprotein A5; FPLC, fast-protein liquid chromatography; TG, triglyceride.

We next assessed the activities of the principal plasma lipases together with plasma FFAs. Pre-heparin plasma activities of LPL and HL were both significantly reduced in mutants relative to WT controls (Fig. 2F, G), and plasma FFA concentrations were correspondingly lower in mutants (Fig. 2H). Together, the apolipoprotein, lipoprotein-profile, and lipase data demonstrate accumulation of TG-rich apoB-containing lipoproteins in the setting of reduced intravascular lipolytic activity in *APOA5* mutant rabbits.

### Correlation of plasma APOA5 with lipid and lipase parameters

The previous group comparisons demonstrate a clear lipid and lipoprotein phenotype in mutants but do not directly address whether this phenotype scales with the residual amount of plasma APOA5 protein. To assess this, Pearson correlation analyses were performed across the combined chow-diet cohort (10 WT plus 9 *APOA5*-mutant rabbits, all sampled at 6 weeks of age; r and 95% confidence intervals reported in Supplemental Fig. S3D–G). Plasma APOA5 protein was strongly and inversely associated with plasma TGs, and positively associated with LPL activity, HL activity and HDL-C (Supplemental Fig. S3D–G). Reciprocally, LPL activity was inversely associated with plasma TGs and positively associated with both plasma FFA and HDL-C, while HL activity was positively associated with HDL-C and inversely with TGs. Notably, when examined within each genotype separately, the correlations were attenuated or absent, suggesting that the observed associations across the combined cohort were largely driven by the genotype-level separation and that a threshold effect may exist for APOA5-mediated regulation of lipase activities and lipid metabolism. Taken together, the magnitude of the lipid and lipase phenotype in APOA5 mutant rabbits largely reflects the genotype-driven reduction in plasma APOA5 rather than a continuous dose-response relationship within the normal physiological range.

### Systemic inflammation and hematologic changes in *APOA5* mutant rabbits

TRLs and their remnants are increasingly recognized as drivers of low-grade systemic inflammation and as activators of innate-immune cells (21, 22, 23). We therefore evaluated whether the chronic dyslipidemia documented previously is accompanied by detectable circulating inflammation in *APOA5* mutant rabbits under chow-diet conditions. At 9 weeks of age, plasma concentrations of IL-1β, TNF-α, and CCL2 were significantly elevated in mutants, whereas IL-6 did not differ between genotypes (Fig. 3A–D). Whole-blood analysis at the same time-point revealed selective expansion of the absolute monocyte, granulocyte, and platelet counts in mutants, together with an elevated plateletcrit (Fig. 4A–I); the total leukocyte count, the absolute lymphocyte count and the relative leukocyte differentials were unchanged. Together, these observations indicate a low-grade systemic inflammatory state in *APOA5* mutant rabbits already on chow.

**Fig. 3:**
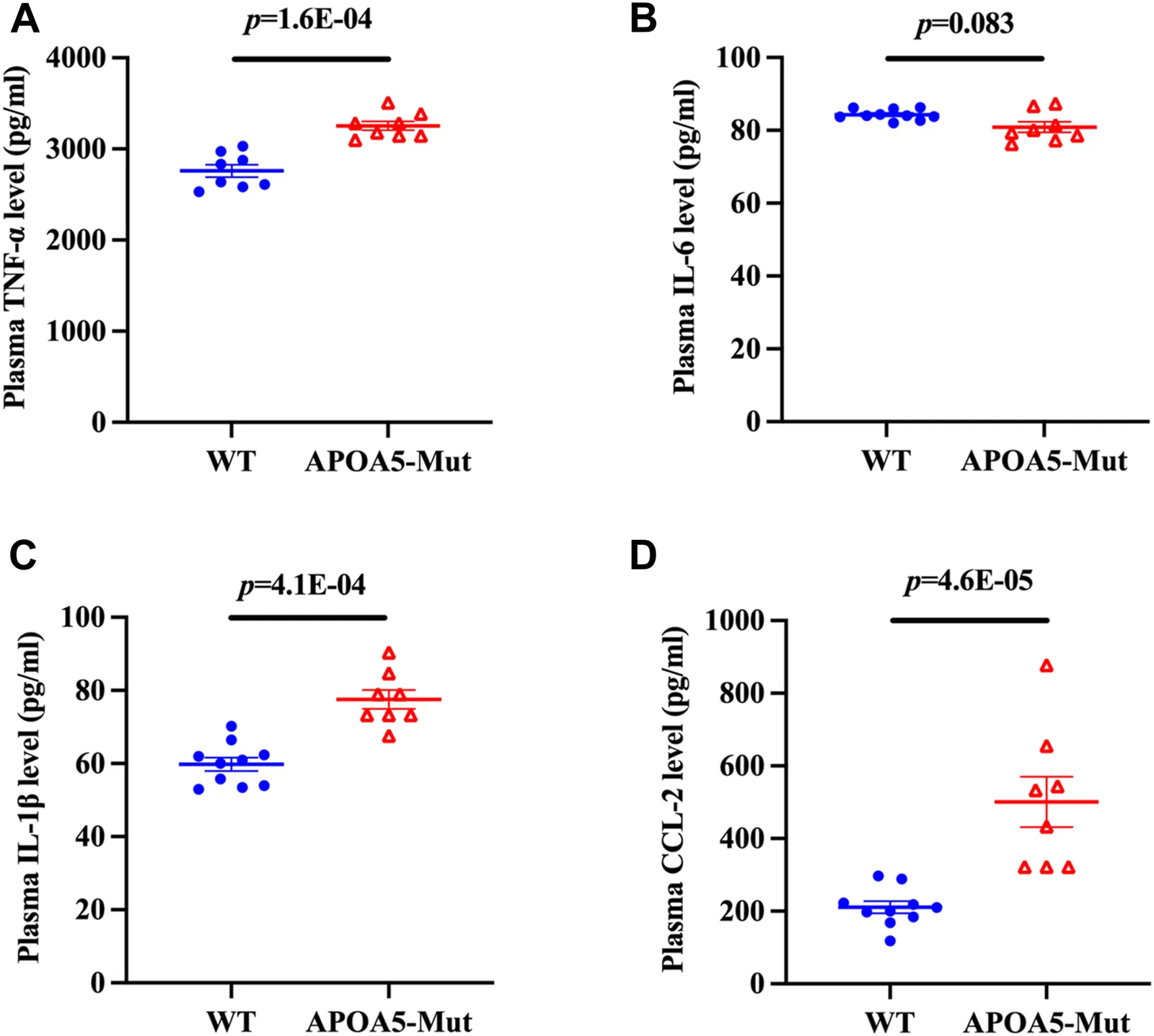
Plasma inflammatory mediators in *APOA5* mutant rabbits at 9 weeks of age. A–D: Plasma TNF-α (A), IL-6 (B), IL-1β (C), and CCL2 (D) measured by rabbit-specific ELISA (WT, n = 10; APOA5-Mut, n = 8). Data are mean ± SEM; statistical tests and exact *P*-values are annotated on each panel. APOA5, apolipoprotein A5.

**Fig. 4:**
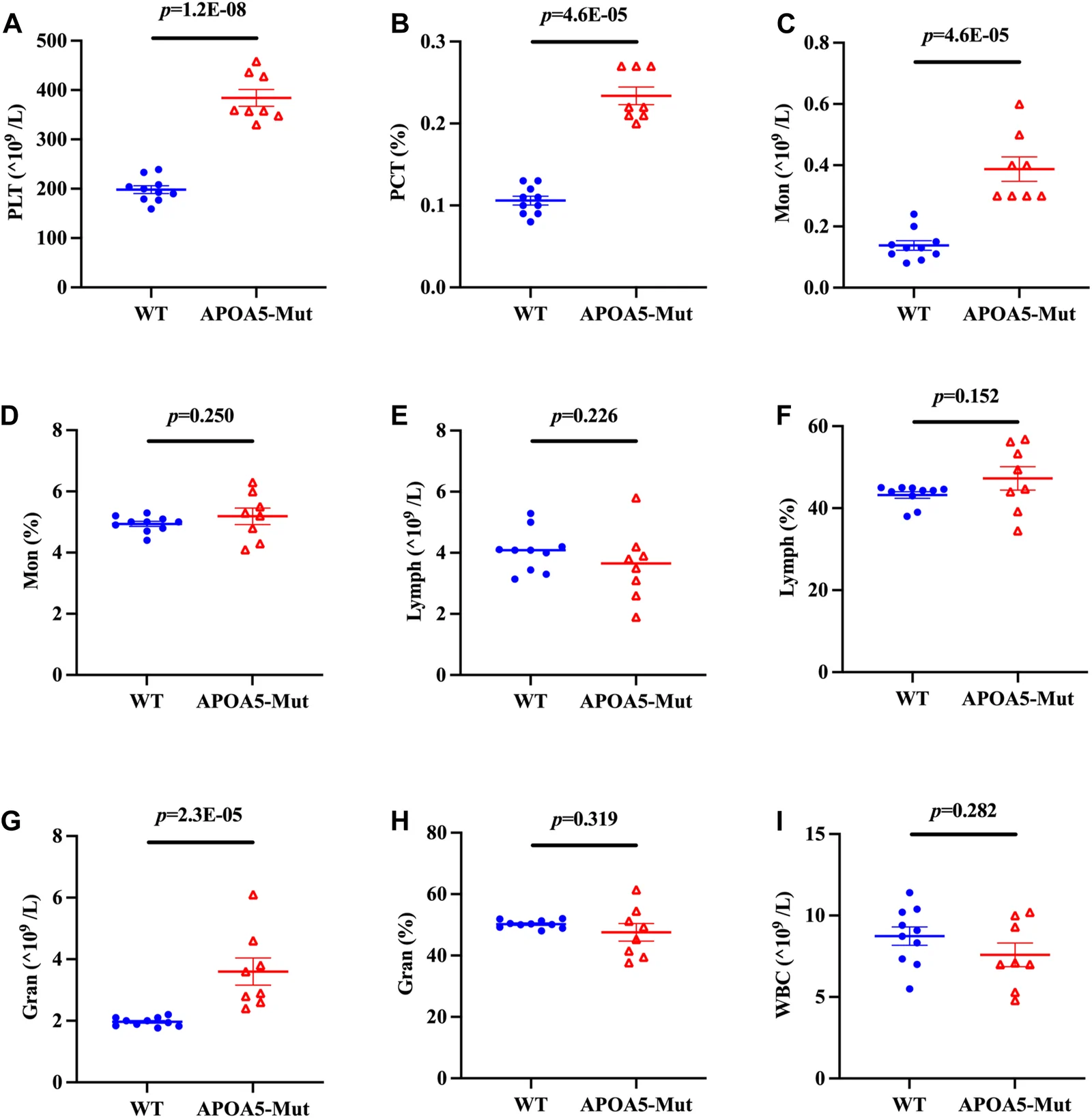
Whole-blood analysis in *APOA5* mutant rabbits at 9 weeks of age. A: Platelet count. B: Plateletcrit. C: Absolute monocyte count. D: Relative percentage of monocytes. E: Absolute lymphocyte count. F: Relative percentage of lymphocytes. G: Absolute granulocyte count. H: Relative percentage of granulocytes. I: White blood cell count. WT, n = 10; APOA5-Mut, n = 8. Data are mean ± SEM; statistical tests and exact *P*-values are annotated on each panel. APOA5, apolipoprotein A5; CCL2, chemokine (C-C motif) ligand 2; IL, interleukin; TNF-α , tumor necrosis factor alpha.

### Aortic atherosclerosis and hepatic steatosis on HFD

Having established that *APOA5* mutant rabbits exhibit a stable atherogenic dyslipidemia, reduced lipase activity and a primed inflammatory state, we asked whether these abnormalities translate into overt vascular and hepatic pathology under a cholesterol challenge. To this end, the 20-week-old cohort was switched to a high-fat, cholesterol-enriched diet for 8 weeks before terminal pathology. Two mutant rabbits (V7 and V8) were excluded from this pathological analysis and reserved for breeding, leaving 10 WT controls and 6 mutants for the terminal assessments.

En face Sudan IV staining of the aortic tree demonstrated extensive atherosclerotic lesion coverage in mutants, contrasting with the more limited disease in WT controls (Fig. 5A, B). Cross-sectional analysis of the aortic root confirmed a markedly larger lesion area in mutants (Fig. 5C, D). The proportion of the lesion occupied by collagen, assessed by Masson's trichrome staining, did not differ between genotypes (Fig. 5C, E), indicating that the enlargement of mutant lesions is not accompanied by a relative gain in fibrous-cap collagen. Immunohistochemistry of the aortic root revealed extensive infiltration of mutant plaques by RAM-11-positive macrophages and α-smooth muscle actin-positive cells (Fig. 5F–H and Supplemental Fig. S4), consistent with both enhanced macrophage recruitment and intimal smooth-muscle cell proliferation. By contrast, the left coronary artery showed no advanced atherosclerotic lesions in either group under the present regimen, although occasional early intimal changes suggestive of fatty streaks were observed in some mutant sections (Fig. 5I); coronary plaque formation in rabbits is known to require more pronounced or more prolonged hypercholesterolemia than was achieved here, either through more aggressive dietary challenge or through use of genetically susceptible backgrounds such as Watanabe heritable hyperlipidemic or *Ldlr*^−/−^ rabbits (13, 14, 15). The combined morphological and immunohistochemical findings therefore indicate that *APOA5* deficiency markedly accelerates the development of macrophage- and smooth-muscle-rich aortic plaques without disproportionate collagen deposition.

**Fig. 5:**
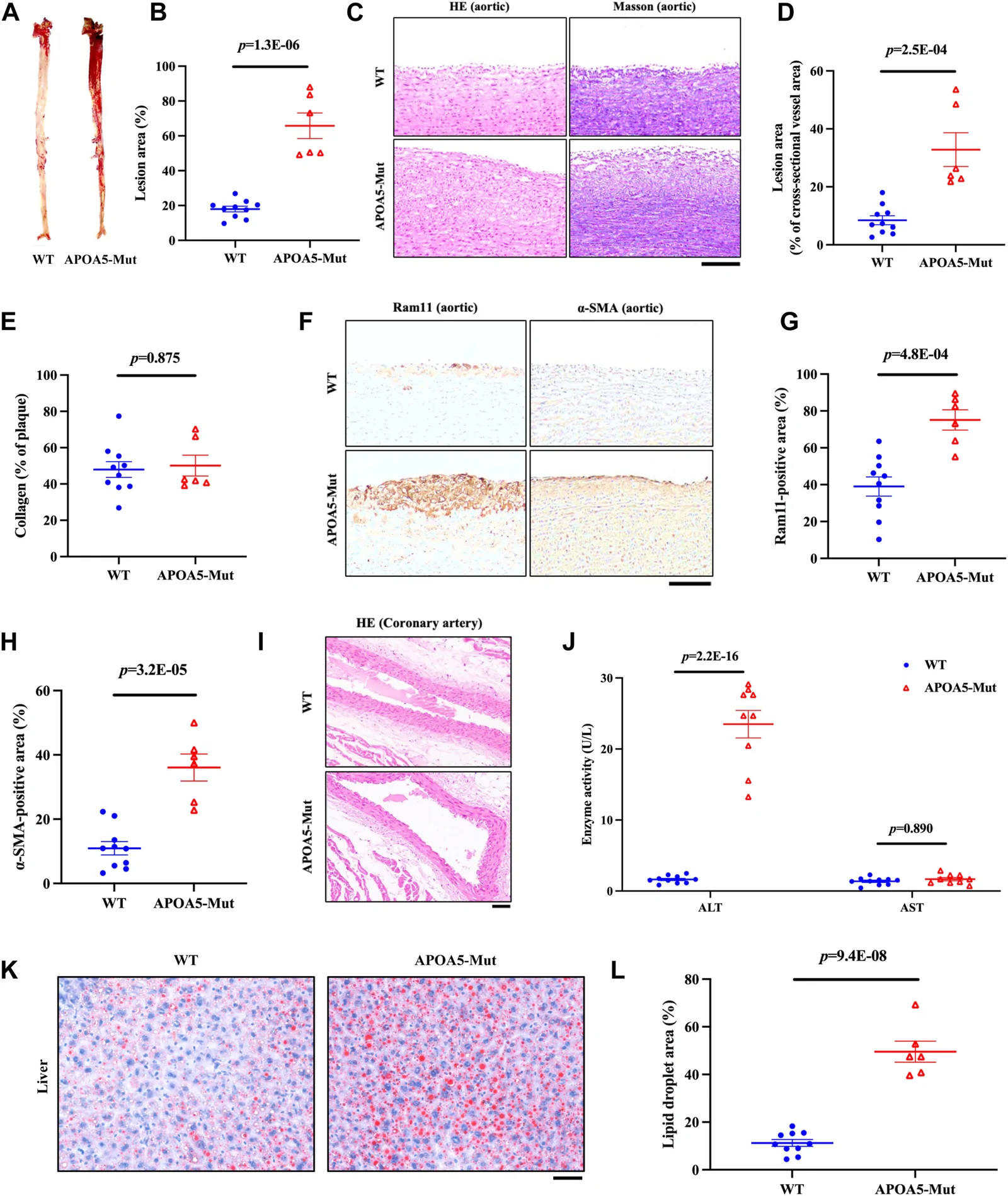
Aortic atherosclerosis and hepatic steatosis in *APOA5* mutant rabbits after 8 weeks of high-fat feeding. A: Representative en face Sudan-IV-stained aortic trees of WT and APOA5-Mut rabbits. B: Quantification of Sudan-IV-positive area as a percentage of total aortic surface (WT, n = 10; APOA5-Mut, n = 6). C: Representative photomicrographs of the aortic root stained with hematoxylin–eosin (HE) and Masson's trichrome (MT). The scale bar represents 100 μm. D: Lesion area as a percentage of total cross-sectional vessel area. E: Aortic plaque collagen area as a percentage of lesion area. F: Immunohistochemical staining of the aortic root for RAM-11 (macrophages) and α-SMA (smooth muscle cells). The scale bar represents 100 μm. G–H: Quantification of RAM-11- (G) and α-SMA- (H) positive area within the lesion. I: Representative photomicrograph of the left coronary artery (HE). The scale bar represents 100 μm. J: Plasma ALT and AST. K: Representative photomicrographs of frozen liver sections stained with Oil Red O. The scale bar represents 50 μm. L: Quantification of Oil-Red-O-positive area. Data are mean ± SEM; statistical tests and exact *P*-values are annotated on each panel. ALT, alanine aminotransferase; APOA5, apolipoprotein A5; AST, aspartate aminotransferase.

Because APOA5 is expressed predominantly in hepatocytes (24), we further evaluated the hepatic consequences of *APOA5* deficiency in the same animals. Even on normal chow diet, plasma alanine aminotransferase was already elevated in mutants relative to controls, whereas aspartate aminotransferase did not differ between genotypes (Fig. 5J). After the HFD challenge, ORO staining of liver cryosections revealed extensive accumulation of neutral lipid droplets in mutants compared with WT controls (Fig. 5K, L). Together with the aortic findings above, these data show that *APOA5* deficiency in rabbits produces extensive aortic atherosclerosis with macrophage- and smooth-muscle-rich plaques alongside marked hepatic steatosis under a moderate cholesterol challenge.

## Discussion

The *APOA5* mutant rabbits described here represent, to our knowledge, the first APOA5-deficient large-animal model and recapitulate three central features of the human APOA5-deficient phenotype. First, the dyslipidemia is qualitatively faithful to human carriers of *APOA5* loss-of-function variants (5): plasma TGs are selectively elevated on chow with a reduction in HDL-C and unchanged total or LDL-cholesterol, and superimposed cholesterol challenge produces severe combined dyslipidemia. Second, plasma FFAs are decreased rather than increased, in agreement with observations in human carriers and in contrast to the elevated FFA reported in earlier *APOA5*-deficient mouse studies (25). Third, the aortic-root lesions are expanded without a proportionate gain in plaque collagen and are heavily infiltrated by macrophages and α-smooth muscle actin-positive cells, indicating an inflammation-rich rather than fibrotic plaque composition.

The mechanistic picture is internally consistent. The magnitude of the dyslipidemia largely reflects the genotype-driven reduction in plasma APOA5 rather than a continuous dose-response relationship within the normal physiological range: lower APOA5 levels are associated with both lower lipase activities and a more severe lipid phenotype, and LPL activity in turn scales with plasma FFA, consistent with LPL-mediated hydrolysis of TRLs being the dominant source of fasting plasma FFA. Considered together with the shift of plasma lipoproteins toward the pre-β region on agarose electrophoresis, the earlier-eluting VLDL peak on FPLC, and the parallel rise in plasma APOB100 and APOB48, these observations are compatible with a model in which APOA5 loss permits the ANGPTL3/8 complex to inhibit endothelial LPL, thereby reducing intravascular TG hydrolysis (6, 7, 8, 9). Independently, work in *APOA5*-deficient mice has shown that loss of APOA5 engages an SREBP-1c-dependent programme of large-VLDL particle secretion (12); the earlier-eluting VLDL fraction we observed in mutant rabbits is consistent with this VLDL-quality change being operative in the rabbit as well, although we did not directly quantify hepatic VLDL secretion and acknowledge this limitation below.

The atherosclerotic phenotype warrants comparison with related rabbit models. In our previous *Apoc3*-knockout rabbit (26), chow-fed animals showed approximately 50% lower plasma TG and resistance to HFD-induced atherosclerosis. The present APOA5-mutant model produces the opposite phenotype, with chronically elevated TG and accelerated atherosclerosis. The two models together delineate the boundaries of the LPL-axis: ApoC3 is a physiological brake on LPL, while APOA5 is the brake-release. This symmetry is supported by drug-target Mendelian randomization data showing that genetically reduced APOC3 protects against cardiovascular disease (27), and by the recently reported Essence-TIMI 73b imaging substudy demonstrating that pharmacological APOC3 antisense inhibition (olezarsen) reduces coronary plaque burden (28, 29). Our rabbit therefore offers a tractable in vivo platform on which the histological consequences of these emerging therapeutics can be tested at the whole-organ level.

Two clinical observations deserve specific mention. (i) Whether *APOA5* mutation predisposes to pancreatitis in this model is unresolved. In humans, *APOA5* loss-of-function variants are an established cause of hypertriglyceridemia-induced acute pancreatitis (2, 30, 31) and the recent *APOA5*-knockout hamster developed acute pancreatitis when challenged with a cerulein protocol (16). We did not perform pancreatic histology or serum amylase/lipase profiling and therefore cannot draw a direct conclusion; we did, however, observe no overt clinical signs of pancreatitis (lethargy, refusal to feed, and abdominal posturing) over the 28-weeks study period, even at TG concentrations exceeding 10,000 mg/dl. Whether the rabbit, unlike the hamster, requires an additional trigger remains a question for future work. (ii) The sex dichotomy in chow-fed TG (males higher than females) and HDL-C (females higher than males) reproduces a biological pattern that has been mechanistically attributed not to a particular lipase per se, but to estrogen-dependent transcriptional regulation of multiple lipid-handling genes (including, in primary human hepatocytes, *LIPC*, *CETP*, *LCAT*, *ABCG5/8*, and *NPC1L1* (19)) and to estrogen-induced LCAT expression that supports HDL maturation (20). Our findings are thus consistent with this broader estrogen-regulated program, rather than with a single lipase-specific mechanism as had previously been suggested.

The study has several limitations beyond the absence of pancreatic readouts. First, hepatic VLDL-secretion was not directly measured by lipoprotein-secretion (Triton WR-1339 or P-407) protocols, and the inferred SREBP-1c engagement is therefore indirect. Second, the 8-weeks HFD regimen produced extensive aortic lesions but only occasional early intimal changes in the coronary arteries; longer-duration or higher-cholesterol challenge will probably be required to reproduce coronary plaque formation in this background (13, 14, 15, 32). Third, we evaluated only one HFD regimen, and the dose-response between APOA5 deficiency and atherosclerotic risk under physiologic dietary patterns remains to be characterized.

## Conclusion

We generated and phenotyped *APOA5*-mutant rabbits using CRISPR/Cas9-mediated targeting of exon 2. Loss of plasma APOA5 protein produces stable chow-diet hypertriglyceridemia and reduced HDL-C, mechanistically linked to decreased LPL and HL activities, an APOB-containing pre-β lipoprotein shift, hepatic steatosis and a primed pro-inflammatory hematologic state. Under 8 weeks of cholesterol-rich HFD, mutants develop severe combined dyslipidemia with extensive aortic atherosclerosis. The model provides a physiologically relevant large-animal platform for the study of residual cardiovascular risk and for the preclinical testing of TG-lowering therapeutics.

## Data availability

The data, analytical methods, and study materials will be available to other researchers for purposes of reproducing the results or replicating the procedure upon reasonable request.

## Supplemental data

This article contains supplemental data.

## Conflict of interest

The authors declare that they have no conflicts of interest with the contents of this article.

## Supplemental data

Supplemental Figures Figure S1: Generation of *APOA5*-mutant rabbits by CRISPR/Cas9 microinjection. A: Schematic of the rabbit *APOA5* locus showing the four exons and three introns. The two sgRNA target sites in exon 2 are highlighted in red and the protospacer-adjacent motif (PAM) sequences in green. B: Summary of three independent microinjection sessions, embryo-transfer recipients, pregnancies, and live births. C: Primers used to assess the five highest-ranked potential off-target sites for each sgRNA. APOA5, apolipoprotein A5; sgRNA, single-guide RNA. Figure S2: Genotype and predicted protein consequence in the 10 founder rabbits. A: Sanger sequencing chromatograms of *APOA5* exon 2 PCR products from founders V1–V10. B: Cloned-and-sequenced genomic alleles from each founder. Deletions are shown as dashes, insertions in red, and substitutions in blue; the resulting size of each deletion (−), insertion (+), or substitution (>) is indicated. C: Predicted amino-acid sequences of the encoded APOA5 mutant alleles. Deletion/insertion-only alleles are shown in gray, frameshift alleles in orange. APOA5, apolipoprotein A5. Figure S3: Sex-stratified analyses and correlation of plasma APOA5 protein with metabolic readouts. A: Plasma HDL-C at 6 weeks of age, stratified by sex within the *APOA5*-mutant group (male, n = 5; female, n = 4). B: Longitudinal plasma HDL-C at 4, 8, 12, 16, 20, 24, and 28 weeks of age, stratified by sex within the *APOA5*-mutant group. C: Longitudinal plasma triglycerides over the same period, sex-stratified. D–G: Pearson correlation analyses of plasma APOA5 protein against plasma triglycerides (D), plasma LPL activity (E), plasma HL activity (F), and HDL-C (G), all measured at 6 weeks of age in the combined chow-diet cohort (WT, n = 10; APOA5-Mut, n = 9). Each panel shows individual animals with a least-squares regression line. Data are mean ± SEM; statistical tests and exact *P*-values are annotated on each panel. APOA5, apolipoprotein A5. Figure S4. Negative antibody control for α-SMA immunohistochemical staining. Left: negative control stained with isotype-matched IgG in place of the primary antibody. Middle: WT group showing positive α-SMA staining. Right: APOA5-Mut group showing positive α-SMA staining. The scale bar represents 100 μm. SMA, smooth muscle actin; APOA5, apolipoprotein A5.
