# Supplementary material for: Hypertriglyceridemia and atherosclerosis in apolipoprotein A5 mutant rabbits generated using CRISPR/Cas9
Source: J Lipid Res. 2026 Sep 22;67(11):101151. doi: 10.1016/j.jlr.2026.101151 (PMC13694806; doi:10.1016/j.jlr.2026.101151)

Fig S1

A

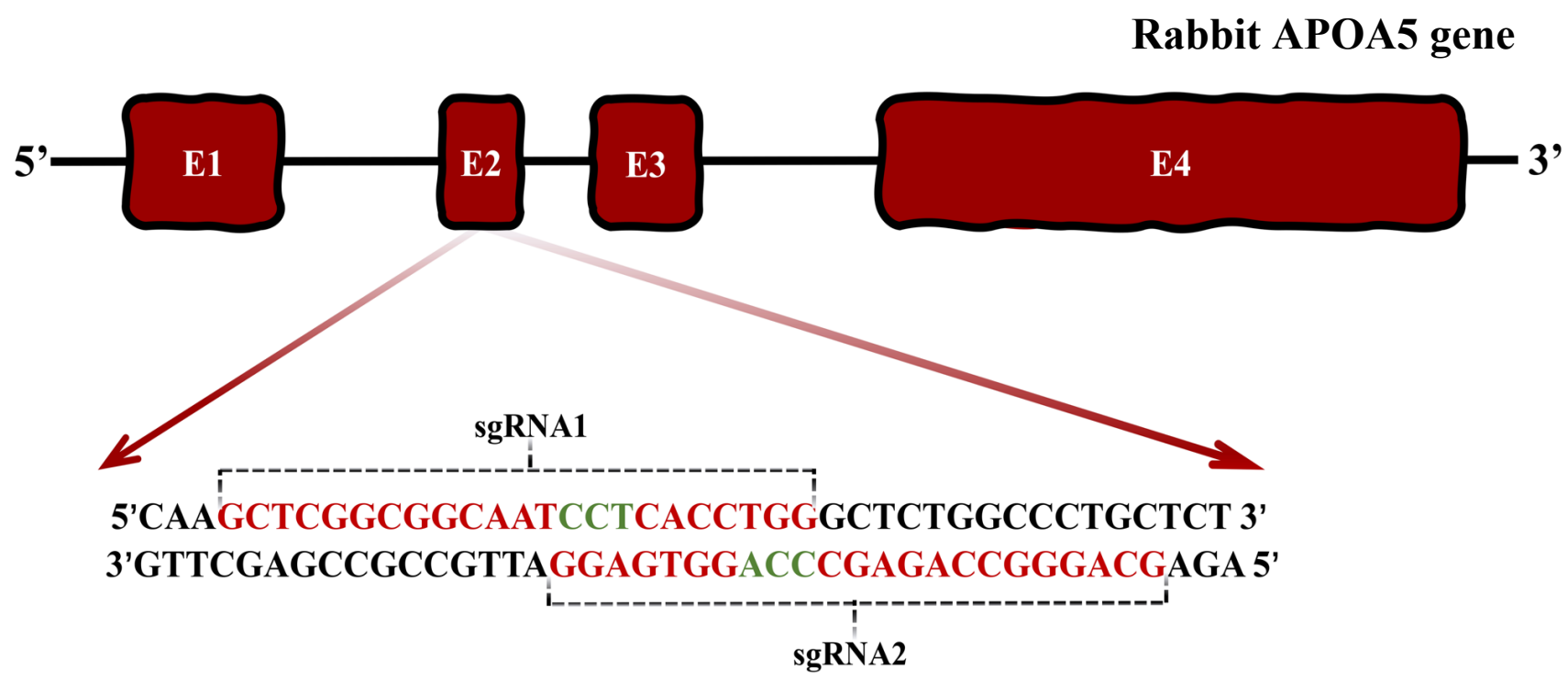

B

Number of microinjection	No.1	No.2	No.3
No. of injected embryos	39	26	39
No. of transferred embryos	29	18	35
No. of recipients	2	1	2
No. of pregnancies	2	0	0
No. of births in total	10	0	0
No. of mutants	10	/	/
Rate of mutations (%)	100	/	/

C

Primers	off-targets of GCAGGGCCAGAGCCCAGGTG AGG	off-targets of GCTCGGCGGCAATCCTCACC TGG
off-targets-1	5′-TCGTCGGCAGCGTCCCTTCATGTGGGGCATCCTT-3′<br>5′- GTCTCGTGGGCTCGGGTCTGAGGATGAGCCGGAAG -3′	5′- TCGTCGGCAGCGTCCCTCTGGTCCTCAGCCTTGA -3<br>5′- GTCTCGTGGGCTCGGAAACATCAGGAGCACGTCGG -3′
off-targets-2	5′- TCGTCGGCAGCGTCCCCAGTGGGTAGACAGCATC -3′<br>5′- GTCTCGTGGGCTCGGTTTCTGTCCATGGTGCTGGC -3′	5′- TCGTCGGCAGCGTCATCGTTCTCCTGACCCACCT -3′<br>5′- GTCTCGTGGGCTCGGTGTTCTCATGAGCGTGTATTTCA -3′
off-targets-3	5′- TCGTCGGCAGCGTCACGGCTGATTTTCTGGCTCA -3′<br>5′- GTCTCGTGGGCTCGGCCCCATATGCTCCCCACATG -3′	5′- TCGTCGGCAGCGTCAAGGGTGCCGAAGTTCATGT -3′<br>5′- GTCTCGTGGGCTCGGGGGTTTGGGCTCAGACTTCT -3′
off-targets-4	5′- TCGTCGGCAGCGTCGTGTCAAAGGCCAGGCTGTA -3′<br>5′- GTCTCGTGGGCTCGGCCTCATCGCCTGTCCCAATT -3′	5′- TCGTCGGCAGCGTCTAACAAGCCACTGCAGGGAG -3′<br>5′- GTCTCGTGGGCTCGGAGCCCACATGCTTTACCCTG -3′
off-targets-5	5′- TCGTCGGCAGCGTCAGGAGGAGCTGAAAAGCCAC -3′<br>5′- GTCTCGTGGGCTCGGCGAGCAGCCATCAGTCACA -3′	5′- TCGTCGGCAGCGTCCCTGGAAAGAGGGAAGCCTG -3′<br>5′- GTCTCGTGGGCTCGGTGGACACGGAGCTATTTGGG -3′

Fig S2

A

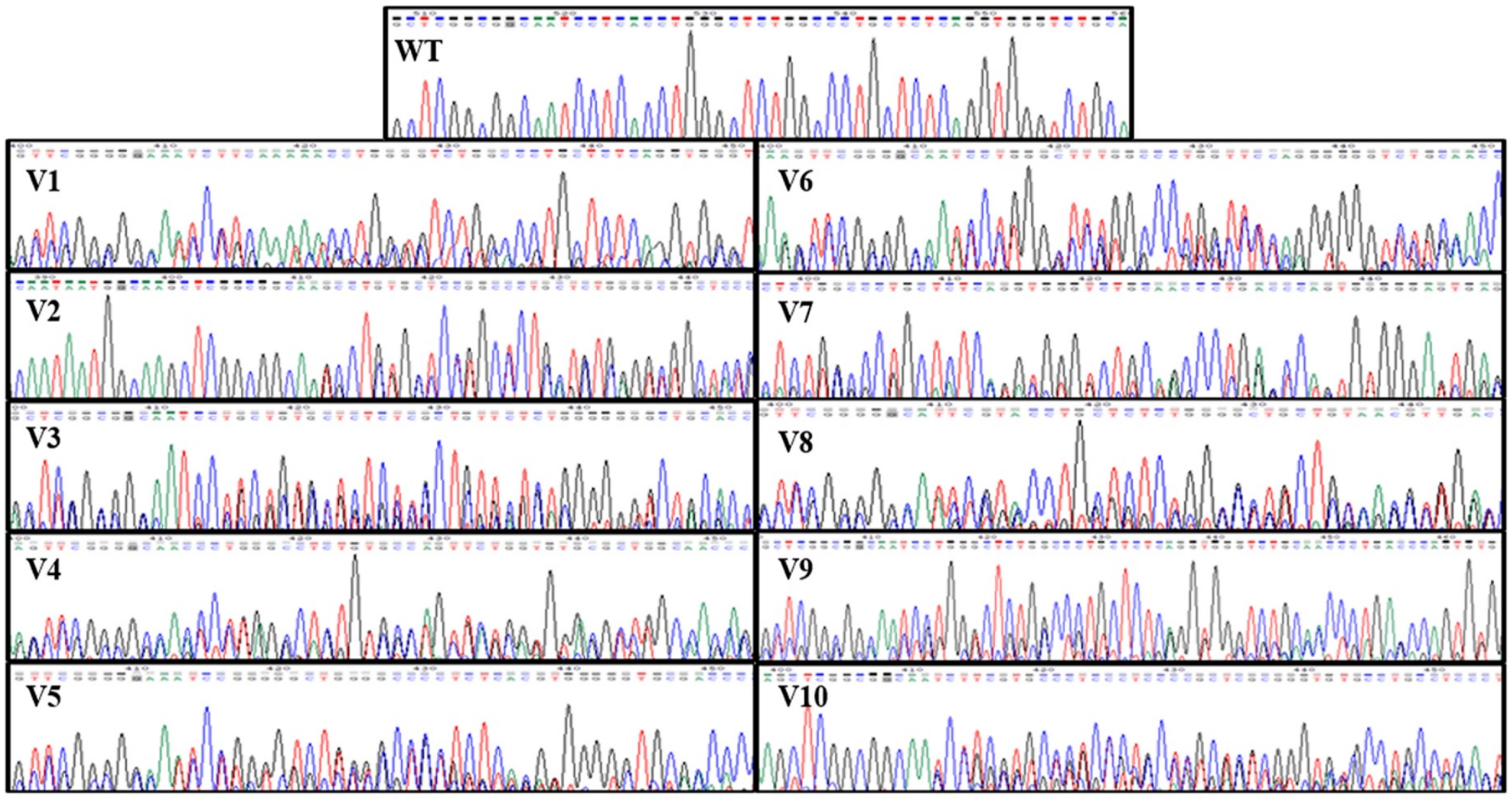

B

WT 5' GGCAAGCTCGGCGGCAATCCTCACCTGGGCTCTGGCCCTGCTCTCAGTGCTTTCAGCCGCC 3'

♂V1
Allele1 5' GGCAAGCTCGGCGGCAATCCTCAAAAACCTGGGCTCTGGCCCTGCTCTCAGTGCTTTCAGC CGCC 3' +4
Allele2 5' GGCAAGCTCGGCGGCAATCCTCGATCATCTCTCTGTCCTCAAACAGTGTCAGGGTCAGCAC GATATCTTCCAGAATGTCCTCGTTTTCCTCATTGTCCAGGAAGTCCTTGTCCTTGATAATTTTC AGCAGATCGTGGTATGTGCCCAGGGAGGCGTTGAACCGATCTTCCACGCCGGAGATTTCCACG GAGTCGAAGCACTCGACCTGGGCTCTGGCCCTGCTCTCAGTGCTTTCAGCCGCC 3' +181

♂V2
Allele1 5'GGCAAGCTCGGCGGCA----------GGGCTCTGGCCCTGCTCTCAGTGCTTTCAGCCGCC 3' -10
Allele2 5'GGCAAGCTCGGCGGCAATCCT-----GGGCTCTGGCCCTGCTCTCAGTGCTTTCAGCCGCC 3' -5

♀V3
Allele1 5'GGCAAGCTCGGCGGCAATCCT-ACCTGGGCTCTGGCCCTGCTCTCAGTGCTTTCAGCCGCC 3' -1
Allele2 5'GGCAAGCTCGGCGGCAATCCT--CCTGGGCTCTGGCCCTGCTCTCAGTGCTTTCAGCCGCC 3' -2

♂V4
Allele1 5'GGCAAGCTCGGCGGCAATCCT-----GGGCTCTGGCCCTGCTCTCAGTGCTTTCAGCCGCC 3' -5
Allele2 5'GGCAAGCTCGGCGGCA---CC 3' -43

♀V5
Allele1 5'GGCAAGCTCGGCGGCAATCCT---CTGGGCTCTGGCCCTGCTCTCAGTGCTTTCAGCCGCC 3' -3
Allele2 5'GGCAAGCTCGGCGGCAATC-------------TGGCCCTGCTCTCAGTGCTTTCAGCCGCC 3' -13

♀V5
Allele1 5'GGCAAGCTCGGCGGCAATCCT---CTGGGCTCTGGCCCTGCTCTCAGTGCTTTCAGCCGCC 3' -3
Allele2 5'GGCAAGCTCGGCGGCAATC-------------TGGCCCTGCTCTCAGTGCTTTCAGCCGCC 3' -13

♂V6
Allele1 5'GGCAAGCTC-----------------------TGGCCCTGCTCTCAGTGCTTTCAGCCGCC 3' -23
Allele2 5'GGCAAGCTCGGCGGCAATCCT-----GGGCTCTGGCCCTGCTCTCAGTGCTTTCAGCCGCC 3' -5

♀V7
Allele1 5'GGC---------------------------TCTGGCCCTGCTCTCAGTGCTTTCAGCCGCC 3' -27
Allele2 5'GGCAAGCTCGGCGGCAATCCT-ACCTGGGCTCTGGCCCTGCTCTCAGTGCTTTCAGCCGCC 3' -1

♀V8
Allele1 5'GGCAAGCTCGGCGGCAATC-------------TGGCCCTGCTCTCAGTGCTTTCAGCCGCC 3' -13
Allele2 5'GGCAAGCTCGGCGGCAATCCT-ACCTGGGCTCTGGCCCTGCTCTCAGTGCTTTCAGCCGCC 3' -1

♂V9
Allele1 5'GGCAAGCTCGGCGGCAATCCT-----GGGCTCTGGCCCTGCTCTCAGTGCTTTCAGCCGCC 3' -5
Allele2 5'GGCAAGCTCGGCGACAA------CCTGGGCTCTGGCCCTGCTCTCAGTGCTTTCAGCCGCC 3' -6, >1

V10
Allele1 5'GGCAAGCTCGGCGGCA----------GGGCTCTGGCCCTGCTCTCAGTGCTTTCAGCCGCC 3' -10
Allele2 5'GGCAAGCTCGGCGGCAATCCTC--CTGGGCTCTGGCCCTGCTCTCAGTGCTTTCAGCCGCC 3' -2

C

WT MASSAAILTWALALLSVLSAAQARRGFWDYFSQSSRDKGREEQSQQQKLVREPVSLKDSLE

♂V1
Allele1 MASSAAIL~~TWALALLSVLSAAQARRGFWDYFSQSSRDKGREEQSQQQKLVREPV~~SLKDSLE -46 +47
KNLGSGPALSAFSRPGTERLLGLLQPEQQGQRQGGAEPAAEAGAGAR
Allele2 MASSAAIL~~TWALALLSVLSAAQARRGFWDYFSQSSRDKGREEQSQQQKLVREPV~~SLKDSLE -46 +106
DHLSVLKQCQGQHDIFQNVLVFLIVQEVLVLDNFQQIVVCAQGGVEPIFHA
GDFHGVEAL DLGSGPALSAFSRPGTERLLGLLQPEQQGQRQGGAEPAAEAGAGAR

♂V6
Allele1 MASS~~AAILTWALALLSVLSAAQARRGFWDYFSQSSRDKGREEQSQQQKLVREPV~~SLKDSLE -50 +42
GPALSAFSRPGTERLLGLLQPEQQGQRQGGAEPAAEAGAGAR
Allele2 MASSAAIL~~TWALALLSVLSAAQARRGFWDYFSQSSRDKGREEQSQQQKLVREPV~~SLKDSLE -46 +44
GSGPALSAFSRPGTERLLGLLQPEQQGQRQGGAEPAAEAGAGAR

♀V5
Allele1 MASSAAIL-WALALLSVLSAAQARRGFWDYFSQSSRDKGREEQSQQQKLVREPGA.
Allele2 MASSAAIWPCSQCFQPPRHGEASGTTSARAAGTKAGRSRASSRSWCGSQEPERQP.

♀V8
Allele1 MASSAAIWPCSQCFQPPRHGEASGTTSARAAGTKAGRSRASSRSWCGSQEPERQP.
Allele2 MASSAAILPGLWPCSQCFQPPRHGEASGTTSARAAGTKAGRSRASSRSWCGSQEPERQP.

♂V2
Allele1 MASSAAGLWPCSQCFQPPRHGEASGTTSARAAGTKAGRSRASSRSWCGSQEPERQP.
Allele2 MASSAAIL~~TWALALLSVLSAAQARRGFWDYFSQSSRDKGREEQSQQQKLVREPV~~SLKDSLE -46 +44
GSGPALSAFSRPGTERLLGLLQPEQQGQRQGGAEPAAEAGAGAR

♀V3
Allele1 MASSAAILPGLWPCSQCFQPPRHGEASGTTSARAAGTKAGRSRASSRSWCGSQEPERQP.
Allele2 MASSAAIL~~TWALALLSVLSAAQARRGFWDYFSQSSRDKGREEQSQQQKLVREPV~~SLKDSLE -46 +45
LGSGPALSAFSRPGTERLLGLLQPEQQGQRQGGAEPAAEAGAGAR

♂V4
Allele1 MASSAAIL~~TWALALLSVLSAAQARRGFWDYFSQSSRDKGREEQSQQQKLVREPV~~SLKDSLE -46 +44
GSGPALSAFSRPGTERLLGLLQPEQQGQRQGGAEPAAEAGAGAR
Allele2 MASSAAPRHGEASGTTSARAAGTKAGRSRASSRSWCGSQEPERQP.

♀V7
Allele1 MA~~SSAAILTWALALLSVLSAAQARRGFWDYFSQSSRDKGREEQSQQQKLVREPV~~SLKDSLE -52 +42
GPALSAFSRPGTERLLGLLQPEQQGQRQGGAEPAAEAGAGAR
Allele2 MASSAAILPGLWPCSQCFQPPRHGEASGTTSARAAGTKAGRSRASSRSWCGSQEPERQP.

♂V9
Allele1 MASSAAIL~~TWALALLSVLSAAQARRGFWDYFSQSSRDKGREEQSQQQKLVREPV~~SLKDSLE -46 +44
GSGPALSAFSRPGTERLLGLLQPEQQGQRQGGAEPAAEAGAGAR
Allele2 MASSAT--TWALALLSVLSAAQARRGFWDYFSQSSRDKGREEQSQQQKLVREPGA.

V10
Allele1 MASSAAGLWPCSQCFQPPRHGEASGTTSARAAGTKAGRSRASSRSWCGSQEPERQP.
Allele2 MASSAAIL~~TWALALLSVLSAAQARRGFWDYFSQSSRDKGREEQSQQQKLVREPV~~SLKDSLE -46 +45
LGSGPALSAFSRPGTERLLGLLQPEQQGQRQGGAEPAAEAGAGAR

Fig S3

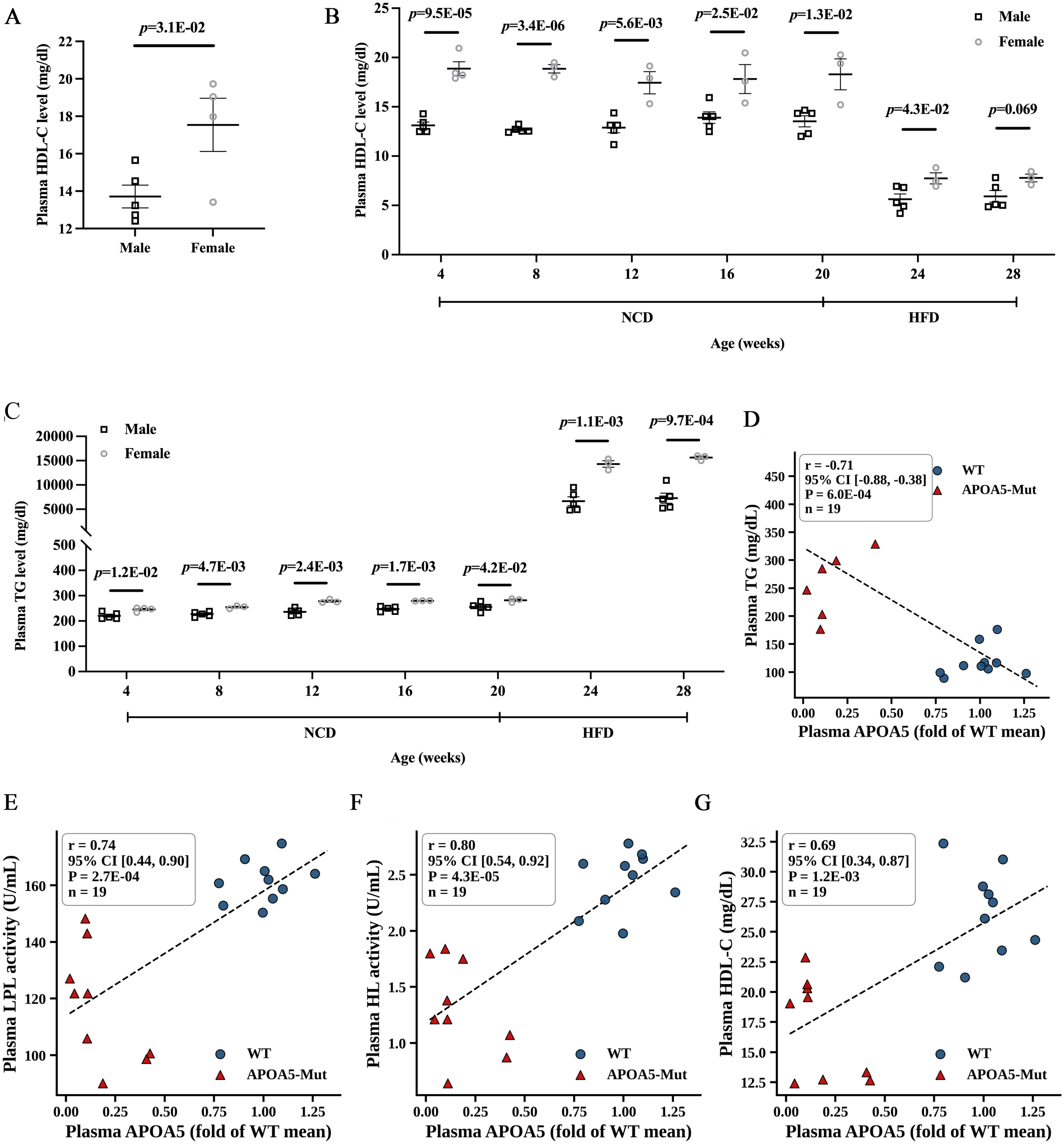

A
p=3.1E-02
Plasma HDL-C level (mg/dl)
Male
Female
B
p=9.5E-05
p=3.4E-06
p=5.6E-03
p=2.5E-02
p=1.3E-02
p=4.3E-02
p=0.069
Male
Female
Plasma HDL-C level (mg/dl)
NCD
HFD
Age (weeks)
C
Male
Female
p=1.1E-03
p=9.7E-04
p=1.2E-02
p=4.7E-03
p=2.4E-03
p=1.7E-03
p=4.2E-02
Plasma TG level (mg/dl)
NCD
HFD
Age (weeks)
D
r = -0.71
95% CI [-0.88, -0.38]
P = 6.0E-04
n = 19
WT
APOA5-Mut
Plasma TG (mg/dL)
Plasma APOA5 (fold of WT mean)
E
r = 0.74
95% CI [0.44, 0.90]
P = 2.7E-04
n = 19
Plasma LPL activity (U/mL)
WT
APOA5-Mut
Plasma APOA5 (fold of WT mean)
F
r = 0.80
95% CI [0.54, 0.92]
P = 4.3E-05
n = 19
Plasma HL activity (U/mL)
WT
APOA5-Mut
Plasma APOA5 (fold of WT mean)
G
r = 0.69
95% CI [0.34, 0.87]
P = 1.2E-03
n = 19
Plasma HDL-C (mg/dL)
WT
APOA5-Mut
Plasma APOA5 (fold of WT mean)

Fig S4

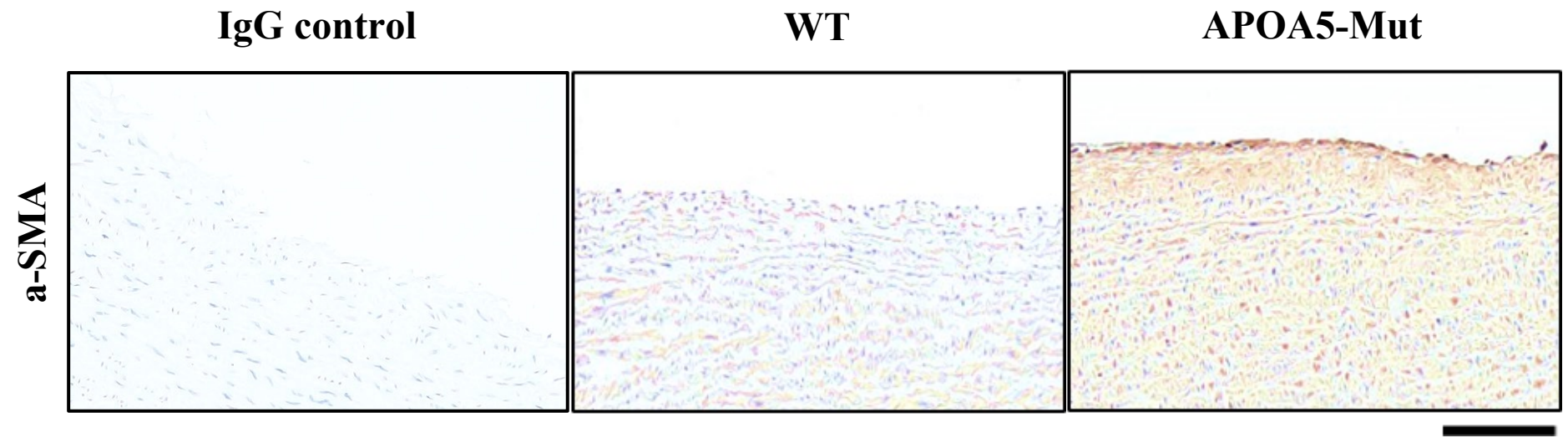

Supplement: Supplementary file 1 [file mmc1.pdf]
